# Screening and managing obstructive sleep apnoea in nocturnal heart block patients: an observational study

**DOI:** 10.1186/s12931-016-0333-8

**Published:** 2016-02-16

**Authors:** Xu Wu, Zilong Liu, Su Chi Chang, Cuiping Fu, Wenjing Li, Hong Jiang, Liyan Jiang, Shanqun Li

**Affiliations:** Department of Pulmonary Medicine, Zhongshan Hospital, Fudan University, Shanghai, 200032 China; Clinical Center for Sleep Breathing Disorder and Snoring, Zhongshan Hospital, Fudan University, Shanghai, 200032 China; Department of Respiratory Medicine, Shanghai Chest Hospital, Shanghai Jiaotong University, Shanghai, 200032 China

**Keywords:** Obstructive sleep apnoea, Heart block, Berlin questionnaire, Continuous positive airway pressure

## Abstract

**Background:**

Nocturnal heart block often occurs in patients with obstructive sleep apnoea (OSA). It is more likely to be undiagnosed in heart block patients who are ignorant of the symptoms of sleep disorder. Berlin Questionnaire (BQ) is a highly reliable way to discover the risk factors of OSA, whereas the validity in sleep-related heart block patients is uncertain. We performed an observational study to address these issues and confirmed the potential protective effect of continuous positive airway pressure (CPAP).

**Methods:**

Patients who were previously diagnosed with nocturnal heart block with R-R pauses exceeding 2 seconds were retrospective screened from the ECG centre of Zhongshan hospital. These recruited participants completed Berlin Questionnaire and underwent polysomnography synchronously with 24-hour Holter monitoring. A cross-sectional analysis was performed to confirm the association between nocturnal arrhythmia and OSA, as well as to assess the diagnostic accuracy of the BQ. Subsequently, subjects diagnosed with OSA (apnoea-hypopnoea index > 5) underwent 3 consecutive days of CPAP therapy. On the third day, patients repeated 24-hour Holter monitoring within the institution of CPAP.

**Results:**

The symptoms of disruptive snoring and hypersomnolence in 72 enrolled patients were more related to the occurrence of nocturnal heart block (*r* = 0.306, 0.226, respectively, *p* = 0.015, 0.019) than syncope (*r* = 0.134, *p* = 0.282) and palpitations (*r* = 0.106, *p* = 0.119), which were prominent trait of our study population. The sensitivity, specificity, positive and negative predictive value of the BQ at a cut-off point of 5 of AHI for detecting OSA in heart block patients was 81.0 %, 44.4 %, 91.07 % and 25 %. Nocturnal heart block does not appear to occur exclusively in severe sleep apnoea. The frequent occurrence of arrhythmias in prominent oxygen desaturation supports the correlation between them. CPAP therapy resulted in significant decrease in the average number of episodes of heart block, from 148.58 ± 379.44 to 16.07 ± 58.52 (*p* < 0.05), same to the change of the longest RR pausing time (from 4.38 ± 2.95 s to 0.57 ± 1.05 s, *p* = 0.169) in 51 patients. The optimal therapy pressure to make the observed arrhythmia disappeared is 12 cm H_2_O.

**Conclusion:**

Concerning high prevalence of OSA in heart block patients, BQ provided an economical and efficient screening method for OSA. For better management, CPAP therapy is feasible to prevent heart blocks avoiding unnecessary concomitant pacemaker implantation.

**Electronic supplementary material:**

The online version of this article (doi:10.1186/s12931-016-0333-8) contains supplementary material, which is available to authorized users.

## Background

Obstructive sleep apnoea (OSA) is an underestimated clinical problem and is widely accepted to be associated with significantly raised cardiovascular morbidity and mortality [1]. Nocturnal heart block often occurs in patients with OSA and is reportedly a consequence of the autonomic effects of recurrent apnoea with subsequent oxygen desaturation, cyclic fluctuations in sympathovagal balance and cardiac hemodynamic changes [2]. As a result, OSA has been identified as a risk factor for almost all types of arrhythmias, ranging from asymptomatic sinus bradycardia to sudden death [3, 4]. An initial study [5] reported that almost 18 % of OSA patients developed bradycardic arrhythmias, while Miller et al. [6] observed that the prevalence of heart block in patients with sleep apnoea was 9–13 %. Screening significant rhythm disturbances assumes particular clinical significance in view of the adverse outcomes [7].

Nearly 80 % of men and 93 % of women with moderate-to-severe sleep apnoea remain undiagnosed within the community [8]. It is more likely to be unrecognized in heart block patients who are ignorant of the symptoms of sleep disorder. The incidence of OSA in patients with nocturnal atrioventricular block or sinus arrest is less defined. Overnight polysomnography (PSG) is the gold standard for OSA diagnosis, but is not generally available and is expensive to perform, requiring highly trained personnel, sophisticated equipment, and an entire night to record data. Berlin Questionnaire (BQ), a reliable method of determining the risk factors of OSA based on symptoms of snoring and daytime sleepiness along with the presence of hypertension or obesity, has good sensitivity ranging from 73 % to 86 %, with a variable specificity of 44 % in sleep laboratory [9] and of 77 % in primary care sites [10]. Considering that not all patients who are observed to have heart block need to undergo PSG, this screening strategy to identify heart block patients at risk of developing OSA should be revalued for validity and feasibility.

It has long been speculated that the vast majority of OSA-related arrhythmias can be reversed during effective continuous positive airway pressure (CPAP) therapy [11, 12]. However, clinical studies on nocturnal heart block patients are limited and conflicting. One randomised controlled trial by Sonya Craig et al. [13] confirmed that there was no significant reduction in frequencies of dysrhythmias comparing therapeutic CPAP groups with the subtherapeutic control, in contrast to the results of another randomized controlled study [14] that confirmed the alleviation of ventricular ectopy by CPAP in patients with heart failure. Moreover, one case report [15] demonstrated that despite atrial pacing, occasional atrioventricular (AV) block still persisted during patient’s follow-up. According to the study of Fietze [16], the prevalence of sleep disorder breathing (SDB) in cardiac pacemaker patients was similar to those without pacemakers. Consequently, screening for sleep apnoea would be necessary before and after a pacemaker. Therefore, we conducted this study to find a simpler method of identifying sleep apnoea in these patients and to examine the effect of CPAP in patients with OSA and heart block.

## Methods

### Patient selection and study design

Firstly, we performed a retrospective screening in patients previously diagnosed with nocturnal bradyarrhythmias in Electrocardiogram Centre of Zhongshan Hospital. As R-R intervals of sleep apnoea-associated heart block often exceed 2 seconds in duration [11], patients whose electrocardiogram (ECG) exhibited R-R pauses exceeding 2 seconds were evaluated for medical history and symptoms. If patients complained of severe palpitation or syncope, they were more likely to have obvious cardiac pathology. These patients were routinely advised to have comprehensive electrophysiological studies to rule out potential life-threatening risks. Patients with a second- or third-degree atrioventricular block type Mobitz II and sinus arrest of at least 2 seconds in duration were included in the study. III° and II° AV conduction block were defined as intermittent or persistent loss of association between P waves and QRS complexes on ECG. Other cardiac bradyarrhythmias were recorded but deemed not to be significant included asymptomatic sinus bradycardia and a sinoatrial block of at least 2 seconds in duration, which were also grouped together as heart block. The exclusion criteria were: 1. chronic atrial fibrillation, atrial or ventricular premature complexes, symptomatic sinus node dysfunction; 2. chronic heart failure, history of stroke, obvious cardiac pathology identified by electrophysiological studies; 3. use of known anti-arrhythmic medication, such as calcium channel blockers digoxin, β-blockers, or atropine; 4. neurological and psychiatric disease; 5. complained of self-reported illness, pain, or circadian sleep disturbances. Those who had other treatment for heart block or received CPAP/bi-level positive airway pressure (BiPAP) also did not meet our inclusion criterion. After application of all the above, we recruited patients that met our criteria to sleep laboratory monitoring for further clinical evaluation.

Then patients consented to undergo polysomnography together with 24-hour Holter monitoring to establish the presence of OSA and arrhythmia. Subjects diagnosed with OSA (apnoea-hypopnoea index > 5) underwent 3 consecutive days of CPAP therapy. On the third day, patients repeated 24-hour Holter monitoring within the institution of CPAP. Our observational study as a part of Australian registered clinical trials was approved by the Ethics Committee of the Fudan University, and appropriate informed written consent was obtained from the subjects.

### Sleep questionnaire and clinical assessment

Subjects with demographic data (age, gender, height, weight, and neck circumference), smoking and alcohol intake history were sampled. Medical history and cardiovascular medication were taking from the recruited patients. Each patient completed surveys including the Epworth Sleepiness Scale (ESS) and an adapted version of the Berlin Questionnaire (BQ). Body mass index (BMI) was calculated as the ratio between weight (kg) and height squared (m^2^). Coronary artery diseases refer to angina, previous myocardial infarction and previous coronary artery intervention. Hypertension, diabetes mellitus and hyperlipidemia are diagnosed according to the new guidelines. The Epworth Sleepiness Scale is an 8-question scale, scored from 0 to 3, that measures excessive daytime sleepiness as a reflection of a subject’s tendency to fall asleep during specific non-stimulating situations. The BQ includes questions regarding daytime sleepiness, snoring, and obesity/hypertension and is used to stratify patients for risk of OSA. The content of the BQ has been previously described in detail by Netzer [10]. The only difference was that 25 kg/m^2^ was used to define obesity instead of 30 kg/m^2^ for all of the included patients were Chinese.

### Polysomnography

The participants who agreed to complete an overnight in-hospital polysomnographic procedure were recorded with an Alice-4 18-channel polysomnograph (Alice-4 Respironics, Pittsburgh, Pennsylvania, USA) in our sleep centre. This investigation included monitoring of the electroencephalogram, electrooculogram, submental electromyogram, electrocardiogram, thoracic and abdominal bands, oronasal airflow by a nasal pressure transducer and an oronasal thermistor, and arterial oxygen saturation by transcutaneous pulse oximetry and a body position electrode. Sleep stages were scored according to the standard criteria of the American Academy of Sleep Medicine. An obstructive apnoea event was defined as the absence of oronasal airflow for ≥ 10 seconds associated with continued or increased inspiratory effort. A central apnoea was defined as the absence of oronasal airflow for ≥ 10 seconds associated with an absent inspiratory effort. Mixed apnoeas were typified by an episode of no air movement resulting from central, followed by obstructive mechanisms. Hypopnoea was defined as a ≥ 50 % reduction in oronasal airflow accompanied by oxygen desaturation more than 4 %. The apnoea-hypopnoea index (AHI) was calculated by dividing the number of apnoeas and hypopnoea by the number of hours of sleep. Interpreters were blinded to the Berlin Questionnaire scores and polysomnographic results, and scoring was performed separately. Patients with an AHI between 5 and 15 were considered to have mild OSA, patients with an AHI between 15 and 30 were considered to have moderate OSA, and patients with an AHI greater than 30 were considered to have severe OSA. In addition to measures of OSA severity, the mean and lowest percentages of oxygen desaturation (taken from the initial diagnostic polysomnography records) were recorded. The oxygen saturation nadir was recorded after all results had been manually checked for technical artefacts.

### Continuous positive airway pressure

According to the guideline of the Chinese Respiratory Society [17], CPAP therapy is best applied for the population diagnosed with modest to severe OSA or mild OSA accompanying obvious adverse symptoms such as cardiovascular diseases. Patients conformed to the indication were performed for consecutive 3 nights. Before the CPAP titration, all patients were given a comprehensive educational explanation of CPAP treatment, and underwent mask fitting from a wide range of mask types and spent 30 minutes acclimatizing to CPAP in a bed during the daytime. CPAP titration was performed following overnight polysomnography using the Autoset self-adjusting CPAP device (REMstar-auto; Respironics). The effective CPAP pressure for each patient was set at the minimum pressure needed to abolish snoring and obstructive respiratory events. In patients for whom significant leak persisted, and who expressed their non-adherence to the given pressure, the pressure was manually decreased in 1 cm H_2_O increments while maintaining the AHI < 10/h and SaO_2_ ≥ 90 % [18]. Acceptable compliance was considered if the patient used CPAP more than 4 h in the third night. Those with the mask leak > 0.4 L/s were recognized as lack of compliance. Compliance was measured the third night results electronically using a SmartCard embedded in the CPAP machine (EncorePro; Respironics).

### 24-hour Holter electrocardiography

Together with overnight polysomnography and the third day on CPAP, 24-hour Holter electrocardiography was performed using a digital recorder with 12 channels. The polysomnography and 24-hour Holter electrocardiography were time-synchronized. Electrocardiograph recordings were automatically interpreted by a software engine and subsequently manually reviewed by 2 researchers. For uncertain classification of an arrhythmia, a cardiologist was consulted.

### Statistical analysis

Categorical data are presented as absolute numbers and percentages and continuous data as the mean ± standard deviation. Differences between groups were compared by one-way ANOVA or the Kruskal-Wallis test (for variables with a non-normal distribution). Correlations were calculated by a Pearson correlation matrix analysis. Simple and multiple logistic regression analyses were performed to identify the risk factors associated with OSA. The association between AHI and heart block was assessed by univariate and multivariate logistic regression analyses with the results reported as an odds ratio (OR) per 1-Uincrease of logarithmically transformed AHI and a 95 % confidence interval (CI). Covariates, such as age, gender, body mass index, diabetes mellitus, and hypertension, were entered into multivariate models if they were significant in univariate models. Patients with CPAP therapy were classified into two subgroups at an AHI cut-off point of 10. Values of *p <* 0.05 were considered significant. Analyses were performed with SPSS 18.0 for Windows (SPSS, Inc., Chicago, Illinois).

## Results

### Characteristics of recruited subjects

**Fig. 1:**
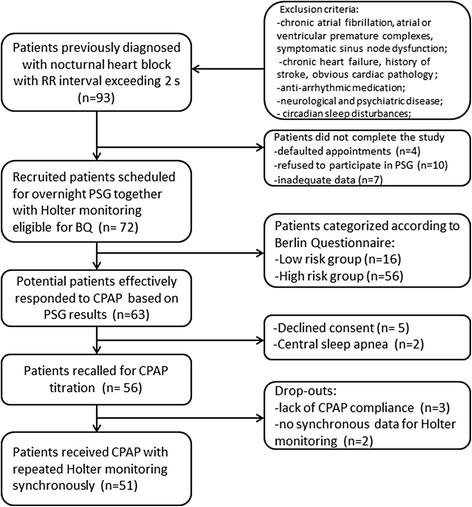
Flowchart of the heart block patients involved in screening and management program of sleep apnoea

From 2008 to 2015, 93 patients previously diagnosed with nocturnal heart block fulfilled the criteria to be enrolled. Four patients missed their appointments and ten refused to participate in the PSG examination. Of the 79 patients who signed consent forms, 7 were excluded because of inadequate data. Thus, standard polysomnography subsequently performed on 72 mainland Chinese patients together with Holter monitoring. Sinus arrest or sinoatrial block was present in 40 patients, sinus bradycardia with complete atrioventricular block was observed in 28 patients, and both sinus arrest or sinoatrial block and sinus bradycardia with complete atrioventricular block were observed in 4 patients. Of the 72 subjects, 2 subjects were diagnosed with central apnoea and the others had obstructive sleep apnoea. Patients were further categorized in predefined strata according to Berlin Questionnaire. Based on standard overnight polysomnography of 72 recruited patients, 63 patients diagnosed with AHI > 5 were potentially responsive to CPAP. Two diagnosed with predominantly central sleep apnoea and 5 patients with declined consent were excluded from CPAP therapy. According to the defined criteria of compliance to CPAP, 3 patients dropped out. Two patients lacked synchronous data of Holter ECG on the third night. Therefore, 56 patients had been called for CPAP titration and only 51 patients finally received CPAP therapy for consecutive 3 nights and repeated the Holter monitoring with the institution of CPAP on the third night. A flowchart of heart block patients included in the screening and managing program of sleep apnoea is displayed in Fig. 1.

The demographics and clinical characteristics of 72 patients are illustrated in Table 1. The high risk OSA group included 56 patients, while the remaining 16 patients were considered to be low risk OSA group according to the BQ. We first investigated the relationship among two groups in terms of age, gender, BMI, and cardiovascular disorders. In general, high risk individuals were older, more obese, and more likely to smoke compared to subjects in the low risk group and also exhibited a higher prevalence of co-morbidities, including hypertension and diabetes mellitus. However, regarding to the occurrence of hyperlipidemia and coronary artery disease, there was no significant difference between the two groups. Patients’ characteristics in cardiovascular co-morbidities also reflected in their medication use. Excessive daytime sleepiness, defined as score > 10, was significantly different between the high and low risk groups (11.34 ± 6.19 vs. 7.07 ± 5.39, *p* < 0.001). In a logistic regression model, after adjusting for other confounding factors, BMI and hypertension were independent predictors in heart block patients for the risks of OSA (OR = 1.4 and 4.8, respectively, *p* < 0.001), while gender and ESS (OR = 0.46 and 1.1, respectively, *p* = 0.16) were not.

**Table 1:** Baseline clinical characteristics according to Berlin questionnaire

Variable	Low risk (*n* = 16)	High risk (*n* = 56)	*p*-value
Demographics			
Age (yr)	48.20 ± 17.34	51.80 ± 13.54	0.185
Female, n (%)	7 (44 %)	4 (7 %)	<0.001
BMI (kg/m^2^ )	23.82 ± 3.36	28.78 ± 7.41	<0.001
Neck circumference (cm)	38.32 ± 2.77	42.20 ± 3.67	<0.001
Medical history			
Smoking status, n (%)	3 (19 %)	38 (67 %)	<0.001
Alcohol, n (%)	2 (12 %)	23 (41 %)	<0.001
Hypertension, n (%)	3 (18 %)	29 (53 %)	<0.001
Diabetes mellitus, n (%)	2 (13 %)	12 (22 %)	0.025 (<0.05)
Coronary artery disease, n (%)	4 (25 %)	8 (14 %)	0.135
Hyperlipidemia, n (%)	7 (44 %)	25 (45 %)	0.897
Causes of deteriorated cardiac conduction			
SA block or sinus arrest, n (%)	8 (50 %)	32 (57 %)	0.618
Complete AV block, n (%)	7 (44 %)	21 (38 %)	0.657
Combined forms of heart block, n (%)	1 (6 %)	3 (5 %)	0.893
Cardiovascular medication			
Platelet aggregation inhibitors, n (%)	1 (6 %)	7 (13 %)	0.490
Diuretic therapy, n (%)	3 (19 %)	18 (32 %)	0.305
ACE inhibitors/ARBs, n (%)	2 (12 %)	19 (34 %)	0.125
Statin therapy, n (%)	2 (12 %)	15 (27 %)	0.241
Symptoms			
Syncope, n (%)	1 (6 %)	7 (13 %)	0.490
Palpitations, n (%)	2 (12 %)	12 (21 %)	0.433
Chest pain, n (%)	1 (6 %)	4 (7 %)	0.903
Snoring, n (%)	10 (63 %)	48 (86 %)	0.039 (<0.05)
Daytime sleepiness, n (%)	8 (50 %)	28 (57 %)	0.618
ESS score	7.07 ± 5.39	11.34 ± 6.19	<0.001
Baseline Holter ECG			
RR interval (>2 s, events/h)	17.14 ± 26.30	196.06 ± 470.05	0.137
Longest RR (>2 s, second)	2.71 ± 0.86	3.56 ± 2.02	0.163
Baseline polysomnography			
Obstructive and mixed apneas, n (%)	0 (0 %)	1 (2 %)	0.597
Obstructive apneas, n (%)	11 (69 %)	49 (88 %)	0.022 (<0.05)
Central apneas, n (%)	1 (6 %)	1 (2 %)	0.345
AHI (events/h)	12.94 ± 7.08	41.3 ± 17.85	<0.001
Mean SaO_2_ (%)	96.14 ± 0.89	91.76 ± 3.90	0.008 (<0.01)
Oxygen saturation nadir (%)	89 ± 5	86 ± 16	0.133

*BMI* Body Mass Index, *ESS* Excessive daytime sleepiness score, *SA block* sinoatrial block, *AV block* atrioventricular block, *SaO*_*2*_ oxyhaemoglobin saturation, *ACE inhibitor* angiotensin-converting enzyme inhibitor, *ARB* angiotensin receptor blocker

Values are presented as mean ± standard deviation or n (%)

The arrhythmias occurring during sleep from the electrocardiogram and synchronous PSG were also analyzed. Although arrhythmias in the high risk group exhibited more severe than those in the low risk group, with regard to the number of episodes of heart block and the longest RR interval time, the differences between them did not reach statistical significance (*p* = 0.137 and 0.163). No significant interactions between the variables of arrhythmia and cardiovascular risk factors (CAD or hyperlipidemia) were observed (*p* = 0.375 and 0.228), but the number of episodes of heart block was a mild predictor of OSA risk (OR = 1.685, *p* = 0.98). Patients usually develop heart block during sleep without diurnal symptoms or signs such as lightheadedness or syncope, on the contrary, these asymptomatic patients presented with characteristics of snoring and daytime sleepiness, with differences between groups that were mild to moderate but statistically significant compared (*p* = 0.039). The symptoms of disruptive snoring and hypersomnolence were more related to the occurrence of heart block (*r* = 0.306, 0.226, respectively, *p* = 0.015, 0.019) than syncope (*r* = 0.134, *p* = 0.282) and palpitations (*r* = 0.106, *p* = 0.119), which were prominent trait of our study population.

### Relationship between severity of OSA and heart block

To investigate the relationship between OSA and nocturnal heart block, we analysed these blocks during the PSG recording. A Pearson correlation matrix analysis was performed in which sleep apnoea severity was significantly related to neither the number of episodes of bradyarrhythmia (*r* = 0.239, *p* = 0.273), nor the RR interval time of second-degree atrioventricular block or sinus arrest (*r* = 0.14, *p* = 0.524). In a second set of analyses with substituted AHI of 30/h, the number of episodes of heart block in patients with AHI ≥ 30/h was not significantly higher than that observed in those with AHI < 30/h (*p* = 0.636), but the RR interval time in patients with AHI ≥ 30/h was significantly longer than that in those with AHI < 30/h (*p* < 0.05). Heart block does not appear to occur exclusively in severe sleep apnoea. Except that 20 % of the patients with an AHI of at least 60/h developed severe arrhythmias during sleep, seven patients with an AHI below 15/h also developed severe arrhythmias, four of which even had more than 40 episodes of long RR intervals.

There is a clear tendency that heart block is more likely to occur with greater O_2_ desaturation. On average, heart block occurred with a mean O_2_ desaturation of 21 %. The inverse association between pronounced O_2_ saturation and prevalence of heart block was, however, not so significantly evident in linear correlation analysis for the night-time recordings (*r* = 0.199, *p* = 0.275).

### Diagnostic accuracy of the Berlin Questionnaire in heart block-associated OSA

Seventy-two patients were further divided into AHI of 5/h, 15/h, and 30/h as cut-off point according to current AASM guidelines, as shown in Table 2. The sensitivity, specificity, and positive and negative predictive values of the BQ in detecting OSA (AHI ≥ 5) were 81.0 %, 44.4 %, 91.07 % and 25 %, respectively. The positive and negative likelihood ratios were 1.45 and 0.43. Considering an AHI of 5/h, 15/h, and 30/h as the cut-off point, the overall diagnostic accuracy (a crude agreement rate) of the BQ was 76 %, 78 %, and 47 %, respectively.

**Table 2:** Diagnostic accuracy of Berlin questionnaire in detecting OSA in patients with heart block

A) Distributions of the two risk groups with respect to AHI								
	Polysomnographic data	High-risk group (*n* = 56)		Low-risk group (*n* = 16)				
	AHI < 5	5		4				
	5 ≤ AHI < 15	17		8				
	15 ≤ AHI < 30	14		2				
	AHI ≥ 30	20		2				
B) Ability of risk grouping with elevated AHI								
	Polysomnographic data	Sensitivity of BQ	Specificity of BQ	PPV	NPV	accuracy	+ LR	**-** LR
	AHI ≥ 5	81.00 %	44.40 %	91.07 %	25.00 %	76.39 %	1.45	0.43
	AHI ≥ 15	89.47 %	35.29 %	60.71 %	75.00 %	77.78 %	1.39	0.30
	AHI ≥ 30	91.00 %	28.00 %	35.71 %	87.50 %	47.22 %	1.26	0.32

*AHI* apnea-hypopnea index, *BQ* Berlin questionnaire, *PPV* Positive Predictive Value, *NPV* Negative Predictive Value, *LR* Likelihood Ratio

### Comparisons of the parameters after the application of CPAP therapy in 51 patients

The patients were evaluated by self-contrast method to assess the efficacy of CPAP therapy as well as the severity of OSA and arrhythmias. 51 patients (47 men, 4 women) were performed for 3 consecutive nights of CPAP treatment in our sleep centre. The mean age of the 51 patients was 58.61 years old (ranged from 37 to 82). The mean AHI was 32.48/h (32.48 ± 20.90) at baseline and 3.65/h (3.65 ± 2.63) on the third treatment night with a mean O_2_ saturation significant improving from 93.62 % ± 3.93 % to 96.32 % ± 1.23 %. The average episodes of heart block during sleep decreased significantly (*p* < 0.05), from 148.58 ± 379.44 before therapy to 16.07 ± 58.52 during CPAP, same to the change of the RR interval time (from 4.38 ± 2.95 s to 0.57 ± 1.05 s, *p* = 0.169). CPAP therapy resulted in the abolition of nocturnal bradycardic arrhythmias in 41 patients (80 %). In the six patients, the episodes of heart block on the third treatment night decreased from 95 % to 73 %. Four patients showed no relevant alterations, one of them even exhibited a 21 % increase in frequency of blocks during CPAP.

When patients were subdivided into AHI (<10) and AHI (≥10) subgroups, there were no significant changes for the group AHI (<10) in O_2_ desaturation, AHI, and arrhythmia parameters. In contrast, the AHI (≥10) group demonstrated great improvement. Table 3 shows the changes in sleep and arrhythmia variables at baseline and after CPAP at a cut-off point of AHI of 10.

**Table 3:** Sleep and arrhythmia parameters in subgroups prior to and following the application of CPAP therapy

Variable	AHI < 10 (*n* = 10)			AHI ≥ 10 (*n* = 41)		*p*-value*
Pressure (cm H_2_O)	8.07 ± 0.15			8.29 ± 3.50		0.647
Compliance (h/night)	4.7 ± 2.5			5.8 ± 3.1		0.307
	Pre-CPAP	Post-CPAP	*p*-value**	Pre-CPAP	Post-CPAP	*p*-value**
Mean O_2_ desaturation (%)	8 %	7 %	0.506	23 %	7 %	0.047
AHI (events/h)	5.28 ± 1.25	3.53 ± 0.35	0.189	41.01 ± 24.20	3.93 ± 2.58	0.014
RR interval(>2 s, events/h)	30.67 ± 38.66	35.33 ± 48.34	0.051	110.89 ± 299.27	16.07 ± 58.52	0.001
Longest RR (>2 s, second)	2.68 ± 1.12	2.6 ± 0.58	0.952	13.81 ± 50.29	0.53 ± 1.00	0.058

Values are given as mean ± SD and n %

*Comparison of CPAP parameters between two subgroups at an AHI cut-off point of 10

**Comparison of changes in the sleep and arrhythmia variables at baseline and after CPAP in two subgroups

**Fig. 2:**
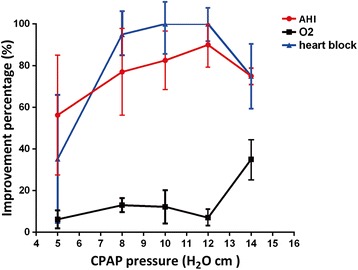
Percentages of improvement in the sleep and arrhythmia parameters at different levels of CPAP therapy pressure. The curve illustrates a dose-associated improvement in O_2_ desaturation, AHI and elimination of arrhythmia at different levels of CPAP therapy pressure (5, 8, 10, 12, and 14 cm H_2_O) in the setting of mild/severe sleep apnoea (AHI > 10). *N* = 5/group

To further analyse the impact of therapy pressure on the parameters of sleep and arrhythmia, we selected different levels of CPAP pressure (5, 8, 10, 12, and 14 cm H_2_O) in mild-severe sleep apnoea group (AHI > 10) and collected corresponding changes of clinical outcomes. The pressure curve (shown as Fig. 2) illustrates a dose-associated improvement in O_2_ desaturation, AHI and elimination of arrhythmia with CPAP therapy in the setting of mild/severe sleep apnoea. The improvement of AHI and O_2_ saturation with CPAP was immediate and evident, even at low levels of CPAP, whereas with an increasing continuous positive airway pressure, the abolishment of arrhythmia events decreased after peaking at 12 cm H_2_O.

## Discussion

The present study observed that respiratory sleep disorders are common among nocturnal heart block patients, especially in patients with high risk factors associated with OSA, such as obesity, habitual snoring or hypertension. To the best of our knowledge, this is the first report of Berlin Questionnaire being administered to nocturnal heart block patients. The principal findings include that BQ is an effective instrument for the recognition of sleep apnoea in heart block patients and this kind of heart block, which occurs frequently during sleep rather than wakefulness in OSA patients, can be largely abolished following the institution of CPAP therapy. OSA appears to be a trigger for heart block during sleep, which may be a potential mechanism for nocturnal bradyarrhythmia.

Previous reports have examined the association between OSA and cardiac arrhythmias in selected group with different severities of OSA or in different populations, such as frequent ventricular premature complexes, conduction delay arrhythmias [3], supraventricular arrhythmias and atrial fibrillation [19]. They also suggested that the incidence of cardiac conduction disorders was more frequent in OSA patients than in the general population [5, 6]. Moreover, cardiac arrhythmias have been documented in up to 48 % of patients suffering from OSA [20]. However, this literature is sparse and conflicting on heart block. A population based study [21] did not observe differences in the occurrence of nocturnal cardiac break among the OSA and matched control groups. Additionally, Harbison et al. [22] only observed 7 cases of nocturnal cardiac break in 45 OSA patients. Compared with other arrhythmias, the Sleep Heart Health Study reported sinus pause was not common in patients with OSA [20]. Little information is available regarding arrhythmia in Asian patients with OSA up to now. In our study all the patients included were mainland Chinese. We performed PSG on them and calculated the occurrence of sleep apnoea as high as 87.5 %, complementing the early epidemiological studies in which a high rate of cardiac arrhythmia patients identified from the general population developed OSA in Asian population. Data from the epidemiological studies in China have estimated that the prevalence of OSA in general population is approximately 3.62–4.81 % [23, 24]. In particular, these characteristics of obesity, disruptive snoring and hypersomnolence were more related to the occurrence of heart block than syncope and palpitations suggested the target population that we emphasized. As a result, the defined population was largely suspected of sleep disorder, which explaining the higher occurrence of OSA. It is notable that those patients conscious of their snoring and sleepiness were more willing to be enrolled in our study. The evaluation and selection from both physicians and patients probably bias the occurrence of OSA in all the arrhythmias but reflect the results in this small kind of arrhythmias. Meanwhile, the high occurrence of OSA, confirmed by PSG, indicates why our study population was most responsive to CPAP.

Regarding the high prevalence of OSA and its potential prognostic implications in nocturnal heart block patients, a combination of clinical symptoms and categorized questionnaires helped to predict its occurrence. The BQ, used as a screening tool, requires a high sensitivity with an acceptable specificity. After systematically performing and examining its validity, the BQ achieved the highest sensitivity at a cut-off point ≥ 30/h for predicting AHI, which is similar to the latest finding of Ulalsi et al. [9]. The performance of BQ in cardiovascular patients has been highly efficient and has a sensitivity of 0.86 and specificity of 0.89 compared with sleep studies [25]. Such results with BQ were also achieved in patients with atrial fibrillation [26] and resistant hypertension [27]. However, the screening instrument in our study was limited by a lack of specificity, as an elevated value may result from snoring. This issue is currently of interest because prior studies showed the occurrence of an atrioventricular block was more likely induced by observed apnoea and nocturnal hypoxemia, independent of snoring. Therefore, without sleep apnoea or hypoxemia, sole snoring hardly affects arrhythmia [28, 29]. This could be a possible reason why the BQ is not a reliable discriminating tool to rule out the non-OSA group. Although BQ is a poor predictor of OSA with a specificity of 0.44, which is valid also for other references [30], that does not mean the Berlin Questionnaire is not recommended as an appropriate instrument to identify patients with sleep apnoea. Given the high cost of sleep monitoring and the lack of dedicated sleep centres, this screening should be considered in the evaluation of arrhythmias with possible risk factors of OSA for its accuracy.

The number of episodes of bradycardia and sinus pauses increased in parallel to the severity of OSA as reported by Roche et al. [31]. Nevertheless, we did not find severity of sleep apnoea assessed by AHI significantly related to bradyarrhythmia, contrary to those who suggested a graded risk for arrhythmias depending on OSA severity [6]. In light of these contradictory results, we must take the large inter-individual variability in the episodes of heart block into account. As an observational study, any confounding risks by unmeasured variables cannot be excluded, even though we had excluded patients with most pre-existing cardiac disease to avoid bias. Our observational study based on nocturnal heart block patients, and was constructed to be neither a fully representative population-based epidemiological study, nor a general arrhythmia-based epidemiological study. Therefore, the sympathovagal predominance, atrial dilatation, impaired baroreflex [32] and hemodynamic fluctuation may predispose subjects to develop arrhythmia. Of note, the influence of O_2_ desaturation on the occurrence of heart block was obvious that supported the correlation between them. Furthermore, through the sub-group analysis at the cut-point of AHI of 30/h, RR interval time in patients with AHI ≥ 30/h was significantly higher than in those with AHI < 30/h as demonstrated in our results, which is consistent with the main conclusions of the previous study [33].

After analysing the features of the subgroup (AHI < 10), we observed that most of the arrhythmia patients with a lack of symptoms were stratified as low risk OSA group, with a minimal or mild residual AHI. It has been hypothesized that a REM-sleep-related AV block occurs independently of episodes of apnoea or hypopnoea. Similar features have been documented in several cases report [34, 35]. Most of them remained irrelevant arrhythmia changes despite CPAP therapy, consistently, the improvement of O_2_ saturation was also not distinct. We concluded that CPAP was not completely preventative in this subgroup, as opposed to those with more severe OSA.

Koehler et al. [36] reported that the magnitude of bradycardia appeared to be related to the severity of hypoxaemia. Several previous studies convincingly demonstrated that the administration of oxygen attenuated the bradycardia [37]. However, a case reported by Ken-ichi Maeno [38] indicated that OSA can provoke heart block without oxygen desaturation. Our findings show the frequent occurrence of nocturnal heart block in prominent oxygen desaturation and provide further evidence that hypoxia with cessation of airflow is a predisposing condition for these arrhythmias. The reversibility of nocturnal arrhythmias by CPAP has been demonstrated in most patients with OSA [39]. In general, the present study shows a favourable outcome with CPAP by abolishing 80 % of nocturnal heart block in 51 consecutive patients, similar to the Becker’s reversal outcomes [40], and an elimination in 72 % of patients of Wolfram’s study [12]. It is well acknowledged that atrioventricular block is observed during both REM and non-REM sleep, but it is hard to differentiate from a REM-sleep–related AV block, because it is often caused by an increased dominated surge of the parasympathetic nervous system. One case report observed that CPAP treatment did not abolish REM-sleep–related AV blocks in patients with mild or no OSA [41], which further supports our subgroup analysis results. Why CPAP could not result in a complete disappearance of nocturnal arrhythmias in several severe patients, irrespective of an obvious cardiac pathology or lack of compliance, remains unknown. After plotting the association of treatment pressure with outcomes, we observed a dose-relative effect on the therapy results. Our study confirms the previous findings [42] in which CPAP application revealed an absence of a sinus and AV block in mild sleep apnoea, which was better explained by pressure effects. Different from an optimal pressure of 15 cm H_2_O to make the observed arrhythmia disappeared as recorded by Ki-Hwan Ji [15], our curve show a pressure of 12 H_2_O cm generates the greatest benefit except for the improvement in O_2_ saturation.

Those symptomatic heart block patients, with lethal risk of long duration of RR pauses exceeding 3 seconds, indeed present a classic indication for the pacemaker implantation according to the current national and international guidelines. In our study, patients whose blocks occurred during nocturnal sleep or daytime nap with risks of sleep apnoea seldom developed long lasting duration of second-degree AV block or sinus block exceeding 5 seconds. A detailed analysis of hourly summary of electrocardiogram result (shown as Additional file 1) and PSG together with Holter recording in a patient (shown as Additional file 2) showed 4957 episodes of heart block with RR interval exceeding 2 seconds during sleep. It has to be noted that these blocks of about 2 seconds duration occurred toward the end of the apnoea episodes, which were abolished largely by CPAP later. This directly demonstrates a strong association between OSA and heart block. A meta-analysis of ten randomized controlled trials has shown that atrial overdrive pacing (AOP) is inferior to CPAP for treating OSA-related heart block and there is a possibility that CPAP therapy may reduce the need for cardiac pacemakers, if patients are screened and treated early. However, the role of AOP in OSA by another meta-analysis was not confirmed [43]. It was reported that SDB was identified in 59 % of those bradyarrhythmia patients implanted with pacemakers [44]. Furthermore, Simantirakis EN et al. [45] indicated AOP was inferior to CPAP on reducing OSA severity. Based on this evidence, those patients with heart block occurring exclusively during obstructive sleep apnoea without obvious pathological abnormality are the best target population for CPAP therapy. Regarding the controversial indication of pacemaker implantation, we emphasize the short waiting time for diagnosis and treatment in our study, since all patients had baseline and follow-up studies just completed within 3 nights. Most often, in clinical practice, after comprehensive evaluation of the medical history, electrophysiological abnormalities and other cardiac symptoms, pacemaker implantation is considered and performed according to the patient’s wish, or based on the recommendation of his or her attending physician.

There should be a low threshold method such as BQ for the identification of OSA that is mandatory to prevent being misdiagnosed by cardiologist or neglected by respiratory physician. Most importantly, this non-pharmacological approach spares patients with concomitant pacemaker therapy. Since patients with long-term pacemaker still exhibit a high prevalence of OSA [44], suggestion of screening OSA is an urgent option that should draw the highest attention of cardiologists.

Several limitations of the present study should be noted. Firstly, lack of electrophysiologic evaluation for underlying disease may obscure the study population. Secondly, it is difficult to match important determinants for arrhythmia patients in a case-control design, owing to inadequate numbers of patient.

## Conclusion

Considering the high prevalence of OSA in heart block patients, a systematic PSG should be ideally offered to patients suffering from nocturnal heart block. Our study complements the previous results that patients with nocturnal cardiac arrhythmia have an increased vulnerability to OSA, and provides a method for systematic OSA detection via BQ screening. For better management in the sleep laboratory, it is feasible to apply CPAP that abolishes the majority of apnoea and apnoea-associated heart block.

## Additional files

Additional file 1:
**Hourly summary of 24-hour Holter electrocardiography showed nearly all these episodes of RR pauses occurred during sleep.** The most severe and frequent episodes of heart blocks appeared at 03:00 am. (TIF 41502 kb) Additional file 2:
**All the recurrent RR pauses in a patient during sleep were not exceeding 3 seconds with longest RR duration 2.7 seconds on Holter monitoring.** Arrows on PSG indicated obstructive sleep apnoea characterized by cessation of nasal air flow and O_2_ desaturation despite continuous thoracic and abdominal respiratory efforts. Occurrence of heart block and sleep apnoea was time-synchronized highlighted by a circle that suggested it was sleep apnoea-related heart block. SaO_2_ = oxygen saturation; ECG = electrocardiogram. (TIF 247130 kb)

## Abbreviations

ACE: inhibitor angiotensin-converting enzyme inhibitor
AHI: apnoea-hypopnoea index
AOP: atrial overdrive pacing
ARB: angiotensin receptor blocker
AV: block atrioventricular block
BQ: Berlin questionnaire
CAD: coronary artery disease
CPAP: continuous positive airway pressure
LR: likelihood ratio
NPV: negative predictive value
NREM: non-rapid eye movement
OSA: obstructive sleep apnoea
PPV: positive predictive value
PSG: polysomnography
REM: rapid eye movement
SA: block sinoatrial block
SaO_2_: oxyhaemoglobin saturation
SDB: sleep-disordered breathing
