# Supplementary figures and images for: Screening and managing obstructive sleep apnoea in nocturnal heart block patients: an observational study
Source: Respir Res. 2016 Feb 16;17:16. doi: 10.1186/s12931-016-0333-8 (PMC4754929; doi:10.1186/s12931-016-0333-8)

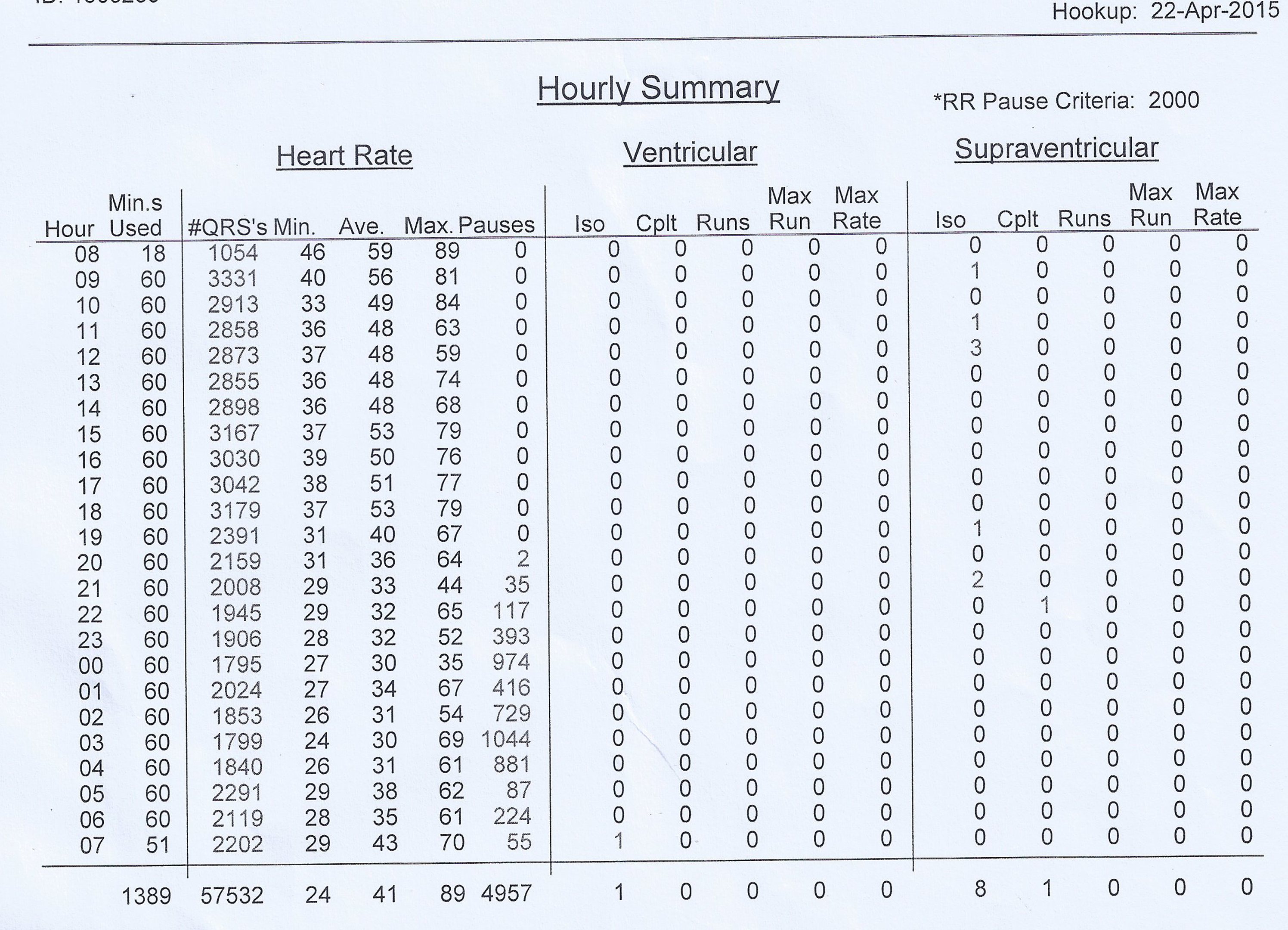

Supplement: Additional file 1: — Hourly summary of 24-hour Holter electrocardiography showed nearly all these episodes of RR pauses occurred during sleep. The most severe and frequent episodes of heart blocks appeared at 03:00 am. (TIF 41502 kb) [file 12931_2016_333_MOESM1_ESM.tif]
